# Is there a subgroup of long-term evolution among patients with advanced lung cancer?: Hints from the analysis of survival curves from cancer registry data

**DOI:** 10.1186/1471-2407-14-933

**Published:** 2014-12-11

**Authors:** Lizet Sanchez, Patricia Lorenzo-Luaces, Carmen Viada, Yaima Galan, Javier Ballesteros, Tania Crombet, Agustin Lage

**Affiliations:** Clinical Research Division, Center of Molecular Immunology, Calle 216 esq 15, Atabey, Havana, 11600 Cuba; National Cancer Registry, 29 y F, vedado, Havana, 10400 CUBA; University of the Basque Country, UPV/EHU, and CIBERSAM, Barrio Sarriena s/n, Leioa, 48940 Spain; Clinical Research Direction, Center of Molecular Immunology, Calle 216 esq 15, Atabey, Havana, 11600 Cuba; Center of Molecular Immunology, Calle 216 esq 15, Atabey, Havana, 11600 Cuba

**Keywords:** Long-term survivors, Survival, Mixture models, Non-small-cell lung cancer

## Abstract

**Background:**

Recently, with the access of low toxicity biological and targeted therapies, evidence of the existence of a long-term survival subpopulation of cancer patients is appearing. We have studied an unselected population with advanced lung cancer to look for evidence of multimodality in survival distribution, and estimate the proportion of long-term survivors.

**Methods:**

We used survival data of 4944 patients with non-small-cell lung cancer (NSCLC) stages IIIb–IV at diagnostic, registered in the National Cancer Registry of Cuba (NCRC) between January 1998 and December 2006. We fitted one-component survival model and two-component mixture models to identify short- and long- term survivors. Bayesian information criterion was used for model selection.

**Results:**

For all of the selected parametric distributions the two components model presented the best fit. The population with short-term survival (almost 4 months median survival) represented 64% of patients. The population of long-term survival included 35% of patients, and showed a median survival around 12 months. None of the patients of short-term survival was still alive at month 24, while 10% of the patients of long-term survival died afterwards.

**Conclusions:**

There is a subgroup showing long-term evolution among patients with advanced lung cancer. As survival rates continue to improve with the new generation of therapies, prognostic models considering short- and long-term survival subpopulations should be considered in clinical research.

## Background

For decades, the primary focus of cancer research was the development of therapeutic interventions to cure the cancer or produce a remission. Success with standard cancer therapy (surgery, radiotherapy and chemotherapy combinations) was mainly limited to early stage tumors. Because of the natural history of cancer, it is relevant to understand if we are witnessing real cures, or just delays in the transition to advanced disease at a given rate [1]. Survival analysis addresses such issues.

The relative survival curve for many cancers will reach a plateau some years after diagnosis, indicating that the mortality among patients still alive at that point is near to the expected mortality in the general population [2]. A straightforward way to identify whether a particular dataset might include a subset of long-term survivors is thus to look at the survival curve to identify the existence or not of such plateau [3]. Another approach is to perform a visual inspection of the hazard function (instantaneous risk of death) plot to look for temporal changes suggesting a “cure” might have been achieved for some patients [4].

In most analyses of cancer survival data, the main outcomes (overall survival and/or progression-free survival) are estimated from conventional methods as Kaplan-Meier and Cox regression models. However, these methods might fail to describe adequately the heterogeneity among cancer patients [5]. To overcome that drawback Boag [6] proposed a two-component mixture model for the analysis of survival data when it is known that a proportion of patients are cured. Such cure models, explicitly model survival as a mixture of cured patients (usually modeled using logistic regression approaches) and non-cured patients (usually modeled using survival approaches).

Many variations of cure models have been proposed and extensively applied. However, the applications have been mainly for patients diagnosed at early stages of cancer [7–11]. Almost all reports have used simulated data or have applied the different models to breast or colon cancer in curable stages.

Exploration of survival data looking for a “cured fraction” has not been extensively applied for advanced cancer, where clinical experience indicates that “cures” are extremely rare or even do not exist. Particularly in lung cancer, without curative treatments for patients in advanced stages, few studies have reported applications of mixture cure models [12].

Recently, and because the advent of biological therapies presenting low toxicity, and targeted therapies, evidences of the existence of a long-term survival subpopulation of patients are beginning to appear, and it is thus relevant to know if this subpopulation represents the tail of the survival distribution that have been shifted towards longer survival by the therapy being administered, or if it represents the existence of intrinsic heterogeneity in the patient population, causing multimodality in the distribution of survival times. If such a chronic evolution subpopulation exists, even in the advanced cancer situation, and some patients live enough to allow the intervention of competing causes of death, it could be convenient to think in terms of long-term survivors or “statistically” cured patients [13].

Finally, it should be noted that the presence of multimodality or mixture distributions in cancer patients could be obscured when clinical trials are the main data source for the analysis, because patients included in clinical trials are by definition selected for reduction of heterogeneity.

In the present paper several parametric survival models and mixture models were applied to an unselected population of patients with advanced lung cancer to look for evidence of multimodality in the survival distribution, and to estimate the proportion of long-term survivors.

## Methods

### Data

The NCRC registers all cancers diagnosed in Cuba [14]. Information within cancer registrations is ascertained from hospital records, diagnostic procedures, pathology reports and death certificates. The estimate of registration completeness at NCRC is 80% [15]. Incident cases of NSCLC reported by NCRC were linked to death records provided by the Cuban National Statistics Office of the Ministry of Public Health.

All adults over 18 years, diagnosed with histological or cytological proven non-small-cell lung cancer (NSCLC) at stages IIIb or IV between January 1998 and December 2006, who were registered in the National Cancer Registry of Cuba (NCRC) with follow-up to December 31, 2010 were eligible for analysis. Of the 6425 eligible patients, 4944 (76.9%) were linked with death records using personal identification number. Due to missing or incorrect identification, 11.2% of patients were excluded from the analysis. The rest of the patients (11.9%) were classified as loss of follow up and were also excluded.

### Modeling approach

For the one component model, the survival function S(t) for the overall population survival time and the hazard, the instantaneous risk of death, were fitted assuming the following parametric models: Gaussian, Log-normal, Weibull and Gamma. Additionally, we fitted a two-component mixture model considering the same distributions adjusted to identify short- and long- term survivors within the advanced lung cancer patients. The survival function for overall population survival time T was expressed as:


Where G(t | μ, σ) is a distribution function. The parameters c_k_, (k = 1, 2), with the restriction that 0 < c_1_ < c_2_ ≤ 1 and c_1_ + c_2_ = 1, are the mixed fractions for the K population. The fractions c1 and c2 can be interpreted as the proportion of short-term and long-term survivors respectively. In the model (μ_k_, σ_k_), are the parameters of the parametric distribution G.

The maximum likelihood estimators of the parameters (c, μ, σ) for the one component or two component mixture models were found by maximizing the likelihood function. We used R v3.0.2 (R Core Team, 2013) for the statistical analyses with the EM algorithm implemented in the “rebmix” library [15] of R (R software; http://www.r-project.org).

### Model selection

We compared the parametric models with the Bayesian information criterion (BIC =, where p is the number of parameters and n is the sample size) to find the most probable model given the data. The model with the smallest BIC value was considered the best fit to the observed data. A BIC difference > 10 between the more complex model assuming two components and the simplest model with only one component was considered as very strong evidence to support the two components approach against the simplest alternative [16].

### Ethics

The use of the data here reported was approved for research purposes by the appropriate Ethical and Research Commitee of the National Cancer Registry of Cuba. Anonymized records (non-patient identifiable data) were provided by the NCRC.

## Results

**Figure 1:**
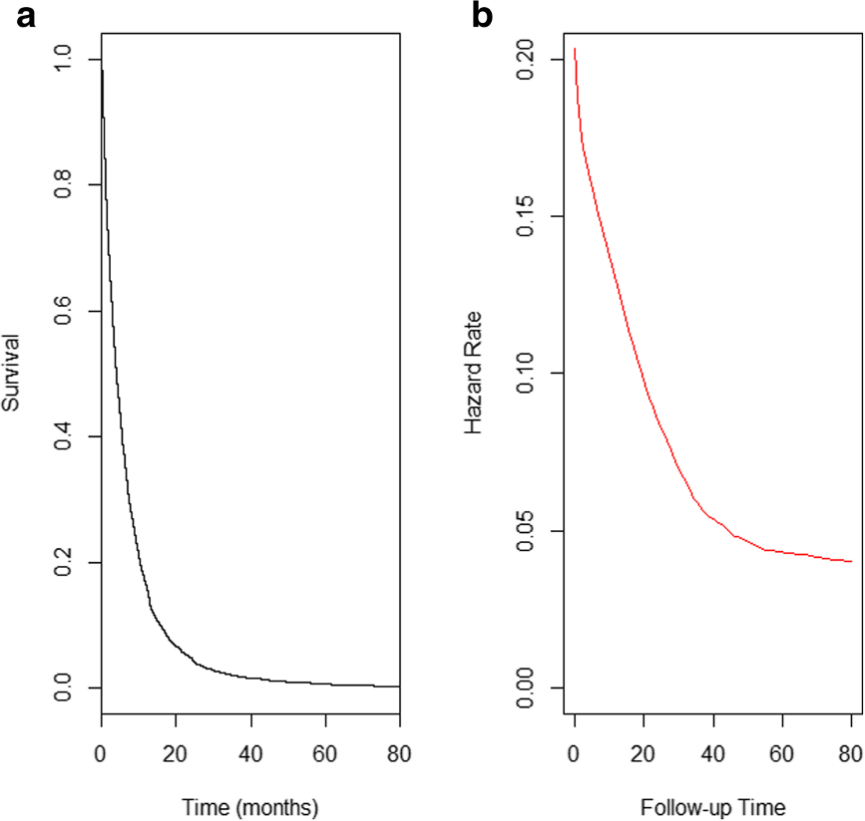
**Cumulative survival a) and hazard curves b) for advanced non-small cell lung cancer registry by the Cuban Cancer National Registry.** 1998–2006.

The median survival time of the Cuban advanced NSCLC patients was 3.93 months. Note that in the survival curve (Figure 1a) it is possible to distinguish a plateau at the end of the study period. Accordingly, the hazard function (Figure 1b) shows a monotonic decreasing curve. Both graphics suggest the presence of two different populations.

**Figure 2:**
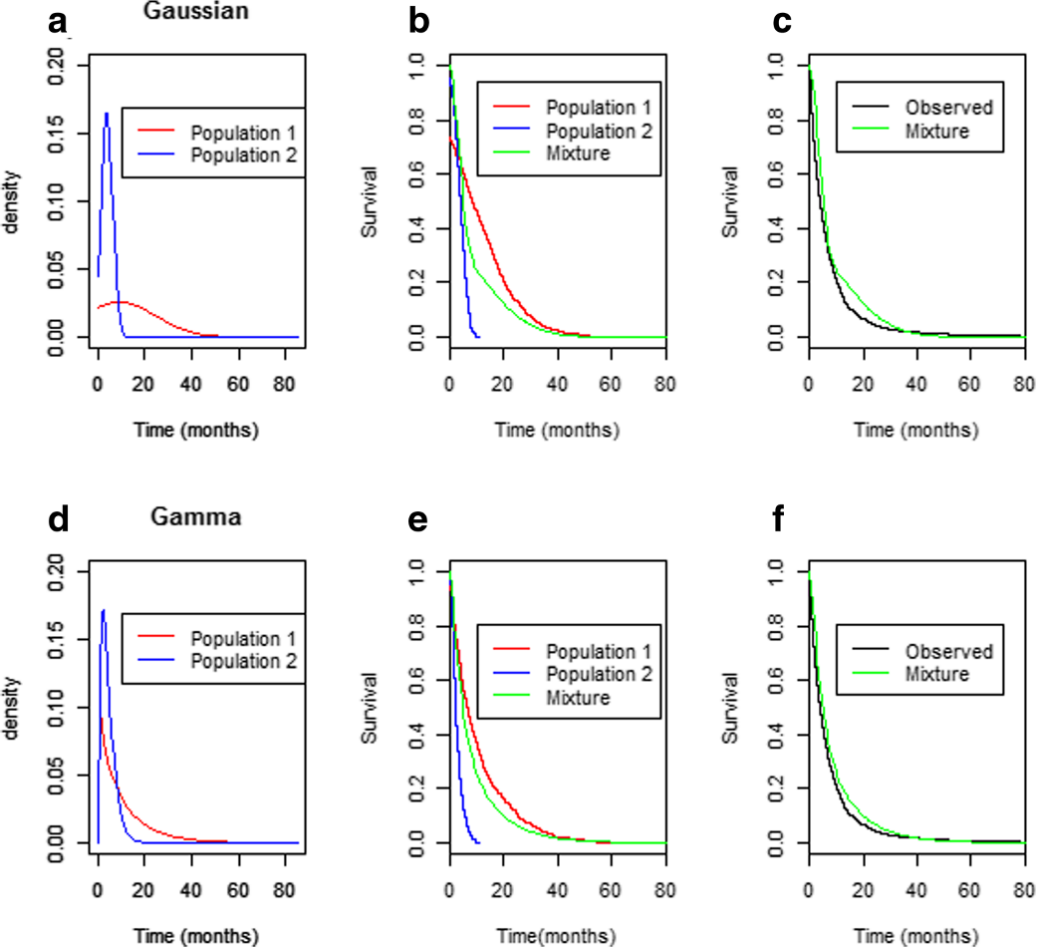
**Illustration of survival patterns of short-term, long-term and mixture populations. a)** Density survival curves assuming Gaussian distribution **b)** Cumulative survival curves for short-term, long-term and mixture assuming Gaussian distribution **c)** Observed vs estimated overall survival assuming mixture of two Gaussian distributions **d)** Density survival curves assuming Gamma distribution **e)** Cumulative survival curves for short-term, long-term and mixture assuming Gamma distribution **f)** Observed vs estimated overall survival assuming mixture of two Gamma distributions.

For all of the selected parametric distributions (Gaussian, log-normal, Weibull or Gamma), the two components model presented the best fit. Gaussian distribution showed the greatest changes in BIC values, while the Gamma distribution provided the best fit to the data (see Table 1). In all models the BIC difference between one- and two-component models was greater than 10, supporting the most complex model and thus the likely existence of two populations of patients. In the Gamma model, the population with short term survival (almost 4 months median survival) represented 64% of NSCLC patients. The population of long-term survivors, which included 35% of patients, showed a median survival close to 12 months.Models assuming Gaussian and Gamma distributions were selected to illustrate the density and cumulative survival curves for short-term and long-term survival populations (Figure 2). Figure 2a and d show the density functions for Gaussian and Gamma distribution respectively. The density peak at 4 months for the first population, indicates that most patients died at that moment. However in the second population the density is flattened. Figure 2b shows no survivors after 11 month for short-term survival population whereas 45% of long-term survival population is still alive. Nevertheless, assuming Gamma distribution (Figure 2e), no patients of the first population are still surviving at month 24, while 10% of long-term survival population died afterwards. As seen, the mixture curves, either for Gaussian or for Gamma distributions, fit quite well the observed survival (Figure 2c, f).

**Table 1:** **Mix fraction and median survival times estimated for short- and long- term survival populations using different parametric models**

Distribution	Numbers of components in the model	Short term survival population		Long term survival population		BIC
		c	Median	c	Median	
Gaussian	One	1	7.21	-		36622.4
	Two	0.80	3.86	0.20	19.9	31353.9
Weibull	One	1	8.27	-		30320.8
	Two	0.92	9.17	0.08	10.10	29528.7
Log normal	One	1	4.52	-		29094.0
	Two	0.77	4.22	0.23	6.57	28942.5
Gamma	One	1	7.25	-		29250.5
	Two	0.64	3.57	0.35	11.9	28610.3

c, Mix fraction in the total population; BIC, Bayesian information criterion. The model with the smallest value of BIC has the best fit.

## Discussion

Is there a subgroup with long-term survival among patients with advanced lung cancer? Our data suggest an affirmative answer. The survival data of advanced NSCLC patients reported by the NCRC could be best explained by a complex mixture model of two populations than for a simpler model assuming only one homogeneous population. In summary, the results provides evidence of the existence of a mixture of populations, including one with long-term survival, consisting of more than 10% of all reported cases, with a survival time greater than 24 months.

Therapies for certain cancer types are believed to induce a subset of long term survivors, such as melanoma [17], breast cancer [18] and multiple myeloma [3]. On the other hand, population based studies have reported the cure fraction estimates for breast [5, 12, 19] and colorectal cancer [13, 20]. However, to our knowledge, this is the first study in an unselected population with advanced NSCLC patients that has found compelling evidence of the existence of a subgroup of patients presenting long-term evolution.

In spite of the fitting complexity of the mixture model, its parameters have a very intuitive interpretation for clinicians. Each subpopulation can be distinguished by two attributes: its size or mix fraction, expressed in percentage; and the corresponding median survival time. It is important to note that estimates of mix fraction can be very sensitive to the parametric distribution chosen to work with. Sometimes, the distribution may not be flexible enough to capture the overall shape of the survival distribution [13]. For this reason, the selection of the parametric distribution to model the observed data should be done carefully. McCullagh and Barry [21] proposed a model selection process algorithm and recommended to fit different distributions to the data to select the best one by using one of the available information criteria.

There are some limitations to both the data and the methodology used in this study. The completeness of NCRC data is known to be high, but may be biased by uncorrected diagnosis dates. Some studies have found this issue to have minimal impact on survival [22]. Stage-specific cure has rarely been estimated due the large proportion of cancer without code of stage in population-based data. Another possible source of bias is that patients without death certificate were excluded from the analysis. As a consequence, under-estimation of survival rates could have happened. However, studies aims to measure that bias, concluded that the effect is minimal when data from population-based cancer registry is used, indicating that the losses can be considered practically random [23, 24]. Furthermore, Yu [20] emphasizes that mixture cure models should be used when there is sufficient follow-up beyond the time when most events occurs. In the case of advanced NSCLC, although estimated median survivals are in the range of 8 to 10 months, several reports [25–27] support the existence of long term survivors - defined as those surviving for more than 2 years after a diagnosis of extensive NSCLC [28].

The transition of advanced cancer to chronicity is a concept that has recently emerged in the literature. Research in cancer treatment has been focused on the search for “cures”, in a naïve extrapolation of the success of antibiotics against infections. This therapeutic paradigm is currently in change driven by the success of modern treatments in prolonging survival in patients with advanced cancer with an ethically acceptable quality of life [29–31], and thus research focus is also moving towards the long term control of the advanced disease. As an analogy worth to note, the history of therapeutic research in Type 1 Diabetes run exactly in the opposite way: whereas it started looking for long term control, and remained so for decades, the therapeutic shift to its “cure” has only become a focus of attention, through the current experimental technologies of pancreatic islet transplants.

Despite their theoretical appearance, these intellectual frames can have huge practical implications for the way clinical research is designed and analyzed. The importance of accounting for long term survivors when the efficacy and safety of immune-oncologic agents is evaluated has been highlighted before [32]. The log rank test and Cox regression models, the standard analyses in immunotherapy evaluation, have maximal statistical power under the proportional hazard assumption. However, Cox models can only provide a satisfactory description of relative survival of the various population groups in the early years after treatment begins, as they cannot present a plateau. Moreover, as survival rates continue to improve, long term survival and cure are becoming increasingly important endpoints when planning oncological clinical trials.

### Further research

Further research is needed to explore the effect of individual prognostic factors and the effect of treatments on the proportion and the failure time of long-term and short-term survival patients. Few current clinical trials have been designed and consequently analyzed with that perspective. Systematic analysis of heterogeneity in survival curves, and of the impact of treatments, not just in the attributes of the survival curves, but on the internal distribution of survival subpopulations, could provide novel and fertile avenues of research.

## Conclusions

This study analysed the survival distribution of advanced NSCLC patients registered in the NCRC. It provides evidence of the existence of a mixture of populations, including a subgroup showing long-term evolution. As survival rates continue to improve with the new generation of therapies, prognostic models considering short- and long- term survival subpopulation should be considered in clinical research. Be able to increase the proportion of patients in the long- term survival group could be a desirable goal for cancer control programs.
